# Pulsed Field Ablation Index–Guided Ablation for Lesion Formation: Impact of Contact Force and Number of Applications in the Ventricular Model

**DOI:** 10.1161/CIRCEP.123.012717

**Published:** 2024-02-23

**Authors:** Luigi Di Biase, Jacopo Marazzato, Assaf Govari, Andreas Altman, Christopher Beeckler, Joe Keyes, Tushar Sharma, Vito Grupposo, Fengwei Zou, Masafumi Sugawara, Atsushi Ikeda, Farshad Raissi, Rahul Bhardwaj, Jonathan C. Hsu, Mark Lee, Rajesh Banker, Sanghamitra Mohanty, Andrea Natale, Qi Chen, Paras Parikh, Xiaodong Zhang, Hiroshi Nakagawa

**Affiliations:** 1 Department of Cardiology, Montefiore Medical Center, Bronx, NY (L.D.B., J.M., F.Z., X.Z.).; 2 Electrophysiology and Cardiac Pacing Unit, Humanitas Mater Domini, Castellanza, Italy (J.M.).; 3 Biosense Webster, Irvine, CA (A.G., A.A., C.B., J.K., T.S., V.G., Q.C., P.P.).; 4 Biosense Webster, Yokne′am, Israel (A.G., A.A., C.B., J.K., T.S., V.G., Q.C., P.P.).; 5 Section of Cardiac Electrophysiology and Pacing, Robert and Suzanne Tomsich Department of Cardiovascular Medicine, Cleveland Clinic, Cleveland, OH (M.S., H.N.).; 6 Department of Cardiology, Nihon University of Medicine, Tokyo, Japan (A.I.).; 7 University of California, San Diego, La Jolla (F.R., J.C.H.).; 8 Loma Linda University Heart Institute, CA (R. Bhardwaj).; 9 Memorial Care Heart and Vascular Institute, Long Beach Medical Center, CA (M.L.).; 10 Hoag Medical Group, Hoag Hospital Newport Beach, Newport Beach, CA (R. Banker).; 11 Texas Cardiac Arrhythmia Institute, St. David’s Medical Center, Austin, TX (S.M., A.N.).

**Keywords:** catheter ablation, electroporation, heart ventricles, swine

## Abstract

**BACKGROUND:**

The effect of contact force (CF) on lesion formation is not clear during pulsed field ablation (PFA). The aim of this study was to evaluate the impact of CF, PFA, and their interplay through the PFA index (PF index) formula on the ventricular lesion size in swine.

**METHODS:**

PFA was delivered through the CF-sensing OMNYPULSE catheter. Predefined PFA applications (×3, ×6, ×9, and ×12) were delivered maintaining low (5–25 g), high (26–50 g), and very high (51–80 g) CFs. First, PFA lesions were evaluated on necropsy in 11 swine to investigate the impact of CF/PFA—and their integration in the PF index equation—on lesion size (study characterization). Then, 3 different PF index thresholds—300, 450, and 600—were tested in 6 swine to appraise the PF index accuracy to predict the ventricular lesion depth (study validation).

**RESULTS:**

In the study characterization data set, 111 PFA lesions were analyzed. CF was 32±17 g. The average lesion depth and width were 3.5±1.2 and 12.0±3.5 mm, respectively. More than CF and PFA dose alone, it was their combined effect to impact lesion depth through an asymptotically increasing relationship. Likewise, not only was the PF index related to lesion depth in the study validation data set (r^2^=0.66; *P*<0.001) but it also provided a prediction accuracy of the observed depth of ±2 mm in 69/73 lesions (95%).

**CONCLUSIONS:**

CF and PFA applications play a key role in lesion formation during PFA. Further studies are required to evaluate the best PFA ablation settings to achieve transmural lesions.

**WHAT IS KNOWN?**

- Pulsed field ablation (PFA) uses nonthermal irreversible electroporation to induce cardiac cell death. The remarkable myocardial selectivity of this new energy source generally leads to well-defined myocardial lesions in histology.
- While contact force is paramount in achieving adequate lesion formation during radiofrequency (RF) ablation, the role of catheter-tissue contact is less clear for PFA.

**WHAT THE STUDY ADDS**

- The aim of this study was to evaluate the impact of contact force, PFA, and their interplay, through the PF index formula integrating both, on the ventricular lesion size in a swine model.
- In this study, contact force and PFA applications proved to play a key role in lesion formation during PFA in a beating swine heart.
- However, further studies are required to evaluate the best PFA ablation settings to achieve transmural lesions even in the atrial chamber.

Pulsed field ablation (PFA) is an emerging method of energy delivery to achieve nonthermal ablation in the management of cardiac arrhythmias. PFA is the result of high electric fields with short pulse delivery to myocytes, resulting in cell membrane permeabilization and nanometric pore formation. Given that the voltage and duration meet a tissue-specific threshold, irreversible hydrophilic pores will form, changing the permeability of the cell, thus ultimately culminating in cell death.

However, the role of contact force (CF) in achieving adequate lesion formation is not clear for PFA. Although some evidence suggested that catheter-tissue coupling alone would be enough to achieve adequate lesion formation,^1^ recent studies have shown the importance of CF on lesion depth during PFA^2^

Therefore, the aim of this study was to explore the role of CF and PFA on lesion size in an in vivo swine model using a multielectrode PFA catheter with a CF sensor (OMNYPULSE, Biosense Webster, Inc) and a bipolar PFA generator (TRUPULSE, Biosense Webster, Inc). Based on CF and the number of PFA applications, a novel parameter—the PFA index (PF index) —was also defined and tested to assess its role in the prediction of the actual lesion size in a swine ventricular heart.

## METHODS

The data that support the findings of this study are available from the corresponding author upon reasonable request.

### The OMNYPULSE Catheter

**Figure 1.**
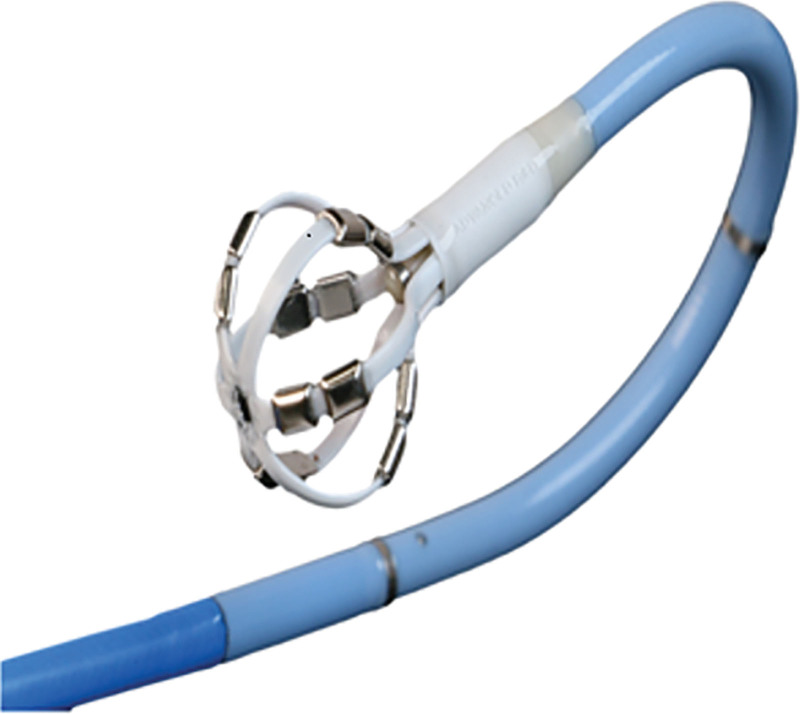
**The OMNYPLUSE catheter. Top**, The OMNYPULSE catheter. The OMNYPULSE is a multielectrode, contact force (CF)–sensing catheter with a distally located spherical cage that is 12 mm in diameter. The catheter has a deflectable tip designed to facilitate electrophysiological mapping of the heart and to transmit pulsed field ablation energy up to 12 electrodes along the catheter tip for ablation purposes. The catheter shaft measures 7.5F and is deployed through a 10F guiding sheath. The OMNYPULSE catheter has CF-sensing technology and incorporates magnetic-based navigation technology allowing the CARTO 3 system to display the location and orientation of the catheter. Images courtesy of ©Biosense Webster, Inc. All rights reserved.

The 12-mm spherical-tip OMNYPULSE catheter possesses 6 splines and 2 electrodes per spline separated by 0.5 mm (Figure 1). The electrodes are active during PFA with no need to choose electrodes or splines during ablation. The location of CF sensor is located just proximal to the basket and the deformation of the spring in the contact sensor allows for measurement of the net magnitude force on the entire basket.

### Animal Description and Procedure Details

The experimental protocol was approved by the Committee on the Use and Care of Animals.

The porcine model is regularly used for preclinical testing for electrophysiology cardiac ablations due to the relative similarities to the human cardiac anatomy. The investigated animals were considered following acclimation and prescreen health assessment.

Before mapping and ablation, the animals were anesthetized with isoflurane and ventilated mechanically and prepared for catheterization per standard procedure. Percutaneous or surgical access of the right femoral vein or artery was performed if needed. PFA ablation was performed under 3-dimensional mapping guidance (CARTO3 system, Biosense Webster, Inc) in both the right and left ventricular chambers. Access to the left ventricle was achieved through transeptal access. For left ventricular mapping and ablation, heparin (10 000–12 000 IU) was administered intravenously with additional doses, as necessary to maintain an activated clotting time >300 s for the duration of the procedure. A baseline intracardiac ultrasound to look for preexisting abnormalities within the heart was performed. Intracardiac ultrasound was also used to guide transseptal access and to detect any test device or procedure-related abnormalities (eg, effusion, thrombus formation). Before ablation, each chamber was mapped using the OMNYPULSE catheter to create a 3-dimensional shell of the right and left ventricles. PFA applications were performed separately (approximately >15 mm) so as not to overlap, thus facilitating gross necroscopy and histopathologic evaluation.

### Study End Points

Two distinct analyses were conducted on 2 different data sets.

1. Part 1: CF and Lesion Dimensions. In this study part, we investigated the relationship between CF and the number of PFA applications on lesion depth and width in the ventricular swine heart. These data were also used as the input to create the PF index equation integrating both the CF and PFA dose values analyzed in this data set (study feasibility or characterization).
2. Part 2: PF Index Evaluation. In this study data set, we assessed the accuracy of the PF index as a quantitative value to represent the ventricular lesion depth in the analyzed swine. For each PF index value, the expected lesion depth was compared with the observed lesion depths (study validation).

### Ablation Protocols

#### Part 1: CF and Lesion Dimensions: Analysis of the Combined Role of CF and PFA Dose on the Ventricular Lesion Size

To avoid a conduction system and ensure lesion size consistency, PFA ablations were applied on the lateral and anterior walls and apex of each ventricle, by avoiding the middle septum and valve leaflets. A distance of >15 mm was required between lesions in the ventricle. Operators were not blinded to ablation parameters, while pathology and lesion assessment were performed blindly to the treatment parameters.

As described in Table 1, the ablation parameters and overall sample sizes were predesignated. Following the designated parameters in each ventricular chamber, PFA ablations were performed in 11 swine (4–7 PFA ablations for each animal depending on size) to achieve a planned number of 120 lesions. PFA was delivered in ×3, ×6, ×9, and ×12 packets of pulses (applications) with a set time delay between them. CF values ranged from 5 to 80 g (Table 1). As the OMNYPULSE catheter possesses a larger tip than a standard 3.5-mm ablation catheter, OMNYPULSE generally requires higher CF to achieve the same pressure per square inch. Therefore, the following CF ranges were considered: (1) low (5–25 g), (2) high (26–50 g), and (3) very high (51–80 g) CFs. For analysis, CF values were allotted to the CF group that was achieved during ablation and regardless of the predesignated target. Higher CF values were specifically targeted in the left ventricle to achieve the deepest lesions.

**Table 1.**
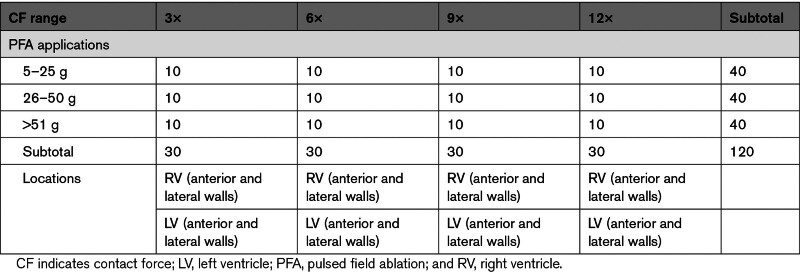
Part 1: Contact Force and Lesion Dimensions. Planned Ablation Numbers per Parameters

The CF and PFA dose values assessed in this data set were also utilized to create the PF index equation based on predefined CF values and PFA doses. The equation relating the PF index to the PFA parameters and CF was empirically created and is shown in the following:

PF index= A*(B(n)*log (n)+C(n))

where A is a constant and B(n) and C(n) are parameters with input variables of applications with n, which is the number of applications or force.

#### Part 2: PF Index Evaluation: PF Index Accuracy in the Prediction of the Ventricular Lesion Depth

In the study validation data set, 72 planned PFA lesions (6 PFA ablation/ventricle) were analyzed in 6 swine. Target ablations in the ventricles were performed through prespecified PF index ranges (300, 450, and 600) and CF parameters (low or 5–25 g, high or 26–50 g, and, finally, very high CF or 51–80 g) and outlined in Table 2.

**Table 2.**
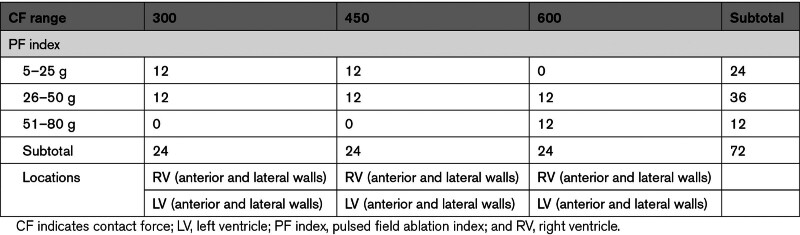
Part 2: PF Index Evaluation. Planned Ablation Numbers per Parameters

Per ablation protocol, PFA ablation was continued for each lesion until the predefined PF index number was achieved regardless of the actual number of pulses delivered. By mathematical definition, the PF index lies on a continuous scale, thus preventing the achievement of the exact prespecified targeted values during ablation. Therefore, PFA ablation was guided by the real-time PF index, and, by using a foot pedal, each operator came off ablation as soon as getting close to the predetermined PF index number. In this regard, lower PF index values and CF thresholds were commonly used within the right ventricle to reduce transmural lesions, which could have prevented accurate measurements of lesion depth at the higher PF index values.

Finally, we investigated whether the targeted PF index could predict the ventricular lesion depth. Therefore, the equation was validated by assessing the observed lesion depth when targeting a specific PF index number of 300, 450, and 600 during ablation. These specific values were chosen to span the relevant dynamic range for the OMNYPULSE catheter and were kept separated to prevent overlap of data sets. The observed lesion depths were then compared with the expected lesion depths generated from the asymptotically increasing equation defining the PF index.

### Gross Anatomy and Histological Assessment

Two hours after the last ablation, before euthanasia, 50 mg/kg of triphenyl tetrazolium chloride was administered intravenously to the animals and further survived the study subjects for 10 to 15 minutes. On termination of the study, animals were euthanized, and gross necroscopy was performed. Histological examination of the ablation lesions was performed using hematoxylin and eosin staining and Masson trichrome staining. At treatment sites, ventricular lesion width and depth were measured based on gross lesion measurement under a Dino-Lite Edge 3.0 digital microscope.

### Statistical Analysis

Continuous variables were presented as mean±SD for normally distributed variables. Median with range (minimum to maximum) was also presented for nonnormally distributed variables. Differences in proportions between groups were tested using the χ^2^ test. Mean values of variables were compared by ANOVA, when appropriate. The association between lesion depth, width CF, PFA applications, and PF index was tested using linear and multiparametric nonlinear regression models. The regression formula was utilized to make a prediction on lesion size from PF index values. Analyses were performed using a significance level of α=0.05 (2-sided). Statistical analyses were performed using MDCalc.

## RESULTS

### Part 1: CF and Lesion Dimensions: The Analysis of the Combined Role of CF and PFA Dose on the Ventricular Lesion Size

Of 120 planned ablations in 11 animals (22 ventricles), 111 could be analyzed (Table S1). Nine lesions were excluded. Five were not found on necropsy due to potential ablation near valvular structures or movement during the ablation, which resulted in a lack of identification; 2 outliers were removed from the 6× PFA data set based on the statistical analysis of the absolute deviation of each lesion per their respective application group; and, finally, 2 lesions were removed due to an incomplete number of applications and because of transmurality, respectively.

Of the 111 analyzed lesions, the average number of lesions/chamber was 5.0±0.9 (range, 4–7) and the amount of PFA applications—×3, ×6, ×9, and ×12 pulses—were kept as equally distributed as possible in the investigated swine. All PFA applications were performed maintaining an average CF value of 32±17 g. As shown in Table 3, similar CF values were maintained across the spectrum of different PFA doses. More specifically, CF values were kept within the low (5–25 g), high (26–50 g), and very high CF (51–80 g) ranges in 40%, 41%, and 15% of animals, respectively. However, due to the difficulty to achieve CF above 50 g in the ventricles, more samples than expected were applied to lower CF ranges as a result.

**Table 3.**
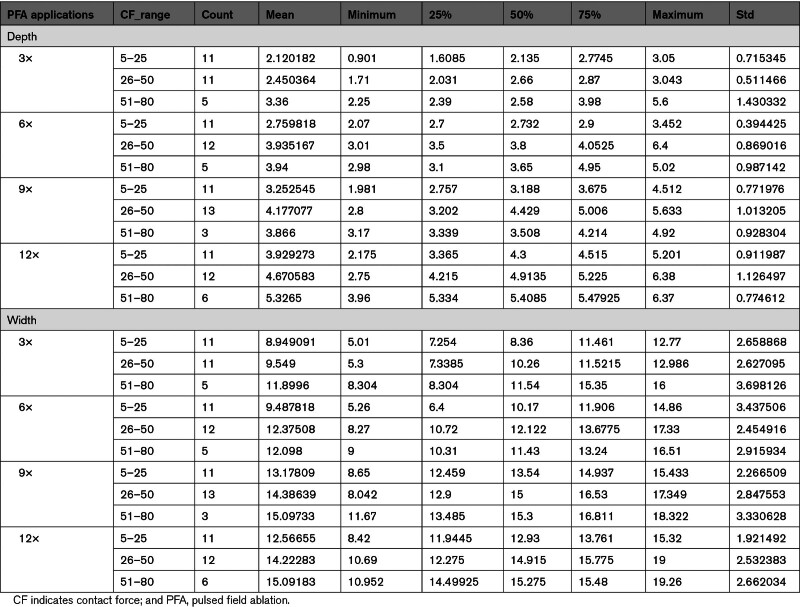
Study Characterization: Correlation Between PFA Dose, CF, and Ventricular Lesion Depth and Width

On necropsy, the average lesion depth and width were 3.6±1.2 and 12.2±3.3 mm, respectively. The lesion depth spanned from 0.90 to 6.4 mm, and ≥5 mm depth was achieved in 17% of the analyzed lesions out of which most (26%) performed with the highest PFA dose (×12). None of the performed lesions was transmural.

In the evaluated data set, the lesion size was related to both the number of PFA applications and the CF values. In fact, as observed in Table 3, the ventricular lesion depth was significantly deeper for low-dose (×3) versus high-dose (×12) PFA (2.48±0.90 versus 4.52±1.09 mm, respectively; *P*<0.005) different from the lesion width. Likewise, regardless of the PFA dose, ventricular lesions performed within the low CF range (5–25 g) were associated with significantly shallower ventricular lesions (2.98±0.98 mm) compared with those performed with high (26–50 g; 3.82±1.21 mm) and very high CF values (51–80 g; 4.23±1.30 mm; *P*<0.05; Table 2).

**Figure 2.**
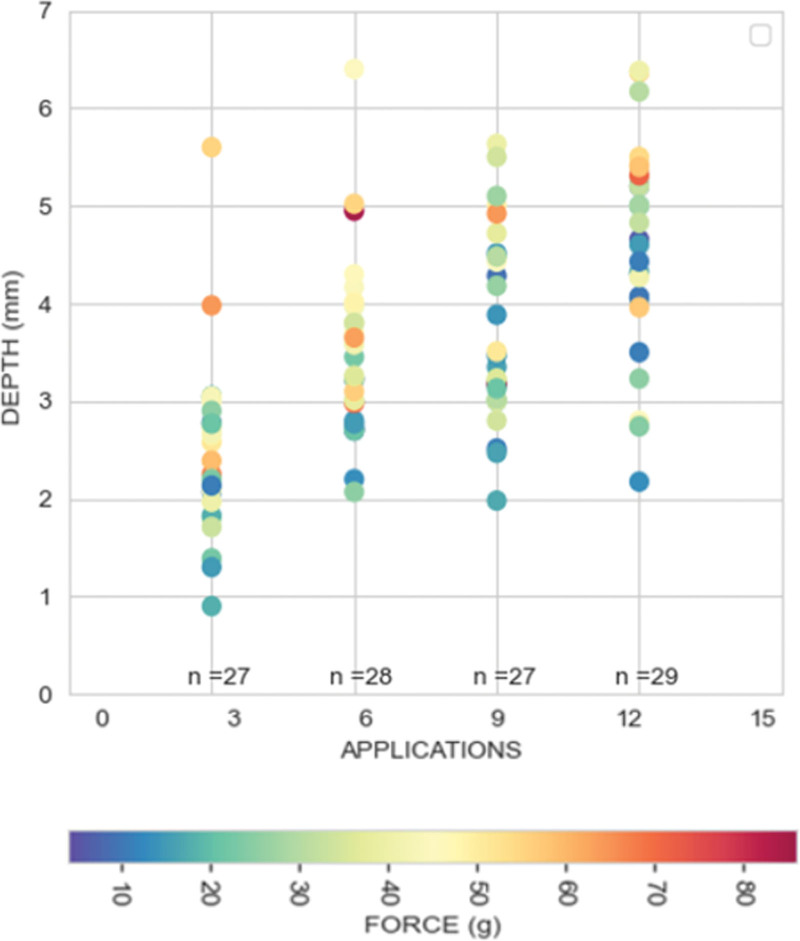
**Study characterization data set.** Impact of contact force (CF) and pulsed field ablation (PFA) on the lesion depth. A significant increase in the lesion size was recorded from very-low-dose PFA (×3) to high-dose PFA (×12) provided that CF >5 g was warranted.

**Figure 3.**
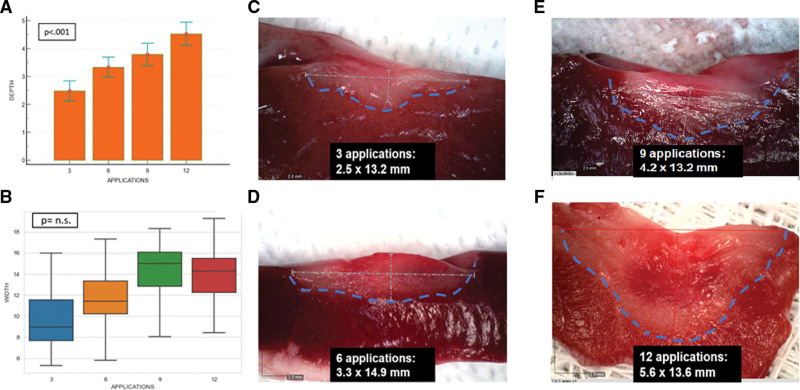
**Study characterization data set.** Impact of contact force (CF) >5 g and pulsed field ablation (PFA) dose on lesion size: histological analysis. Once a good catheter-tissue contact was achieved (CF >5 g), a progressively higher PFA dose was associated with deeper lesions (**A**) and increasing width up to 9 applications at which it plateaus. (**B**). The histological sections were consistent with this observation (**C–F**). In fact, when the system was toggled from ×3 to ×12 PFA dose, despite a slight increase in the lesion width, the average lesion depth nearly doubled. Images courtesy of © Biosense Webster, Inc. All rights reserved.

Regression analyses were then performed to rule out any potential confounding effect of CF and PFA dose on lesion size. To perform this analysis, CF >80-g data were not considered due to the low sample size in each cohort. By regression analysis, we observed that while the relationship between CF and lesion depth (y=2.882+0.0209x; r^2^=0.08; *P*=0.0024) and CF and lesion width (y=10.633+0.0422x; r^2^=0.04; *P*=0.0043) were weakly linearly related, the combination of the PFA dose with the CF applied for each lesion proved to have a greater impact on the ventricular lesion depth through an asymptotically increasing relationship (Figure 2). However, the role of CF and PFA dose on lesion width was less predictable. In fact, differently from lesion depth (Figure 3A), greater PFA applications applied with CF>5 g led to a slight increase in lesion width up to 9 applications at which it plateaus (Figure 3B). A histological example of this kind is reported in Figure 3C through 3F where deeper lesions were observed for high-dose PDA (×12) versus low-dose PFA (×3) despite similar lesion width (Figure 3C–3F).

### Part 2: PF Index Evaluation: PF Index Accuracy in the Prediction of the Ventricular Lesion Depth

As extra lesions were added to increase the sample size in the high CF range (Table S2), 73 PFA lesions were analyzed in 6 pigs to understand whether the PF index (300, 450, and 600 ranges) could predict the actual lesion depth. Due to variability in pig sizes and location of ablation and CF achieved in the ventricles, a slight difference between predefined and achievable CF values was observed. The mean number of PFA applications and CF values associated with each PF index strata is reported in Table S3.

Of the 73 analyzed lesions, 6.8±1.2 and 5.3±0.8 PFA lesions were assessed in the right and left ventricles, respectively. Twenty-nine (34%) were within the 300 (307±21; range, 268–343), 23 (31%) in the 450 (460±24; range, 424–554), and, finally, 21 (29%) in the 600 (547±34; range, 475–590) PF index strata. Of note, the intended PF index values of 600 remained unattainable and resided within the 550 range. As for CF values, 29 (40%), 30 (41%), and 14 (19%) lesions were performed within the low (5–25 g), high (26–50 g), and very high (51–80 g) CF strata, respectively. In this data set, the average lesion depth was 3.6±1.0 mm (3.2±0.9 and 4.2±0.9 mm for the right and left ventricles, respectively) ranging from 1.7 to 6.6 mm and achieved with a mean PF index of 424±105.

Table 4 displays the average lesion depth associated with each attained PF index (ie, 300, 450, and 600) and stratified according to the low, high, and very high CF data sets.

**Table 4.**
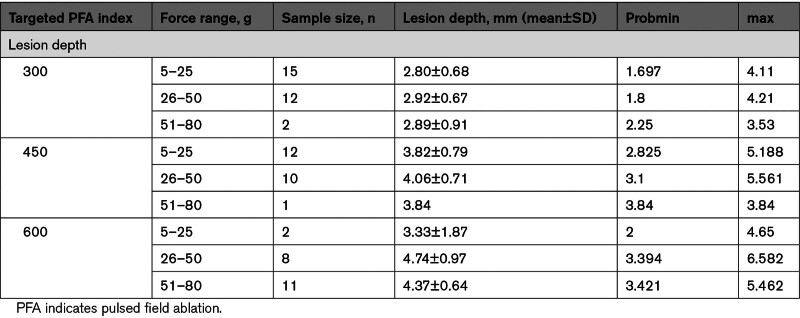
Study Validation: Correlation Between PFA Dose, Contact Force, and Ventricular Lesion Depth

**Figure 4.**
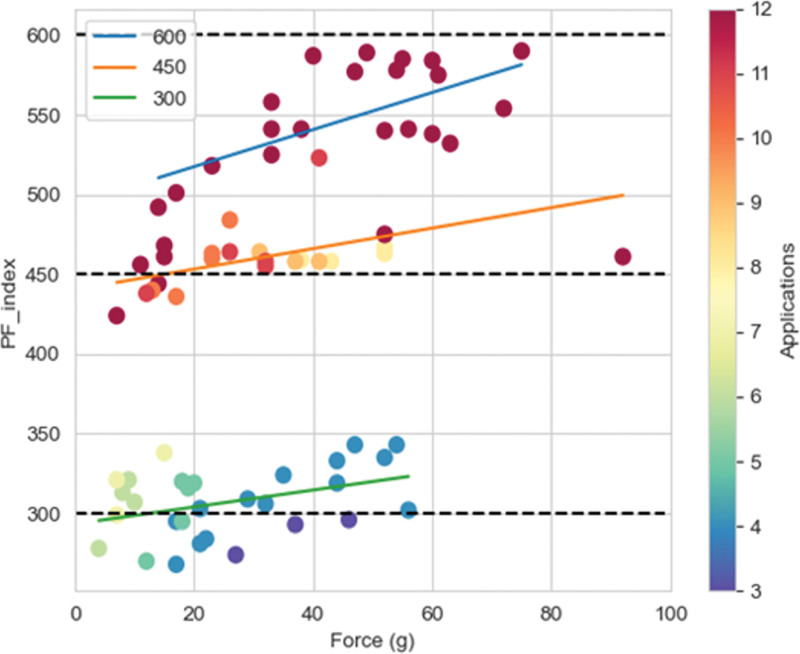
**Study validation data set: relationship between contact force (CF) and pulsed field ablation index (PF index).** PF index and CF were linearly related for each attained PF index value.

**Figure 5.**
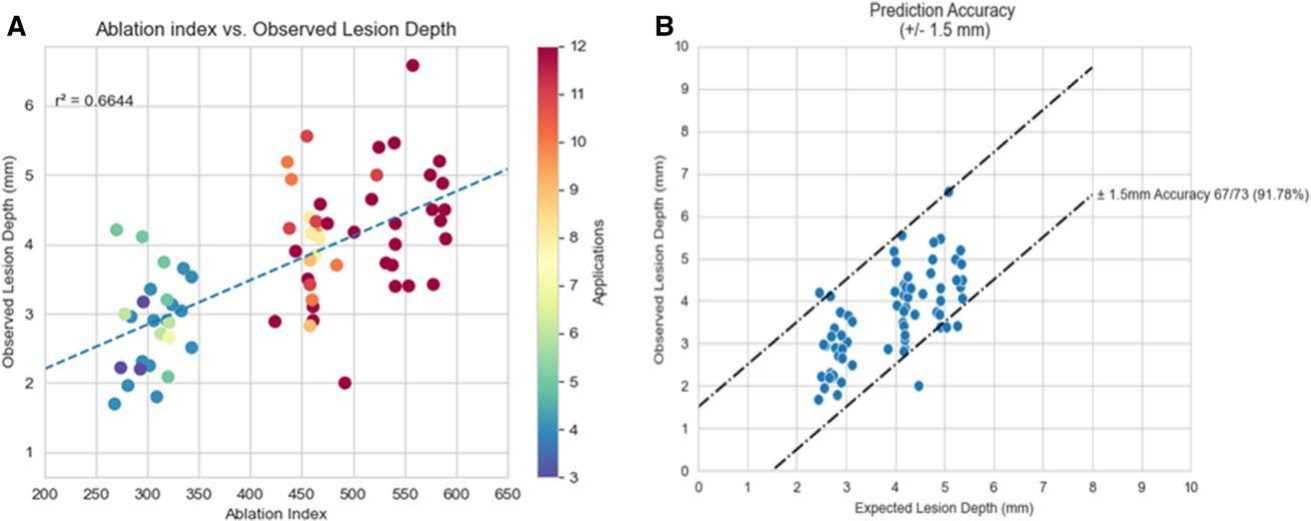
**Study validation data set: correlation between lesion depth and pulsed field ablation index (PF index). A**, The attained PF index values were plotted against the observed lesion depth on histology. The 2 variables were linearly related, and >60% of the variation of the lesion depth was explained by the variation of the PF index parameter (r^2^=0.6644). Furthermore, when the actual lesion depth was plotted against the expected lesion depth, or PF index/100, it was clear that the actual lesion depth on histology was predicted by the PF index values attained during ablation with a prediction accuracy of ±2 mm.

**Figure 6.**
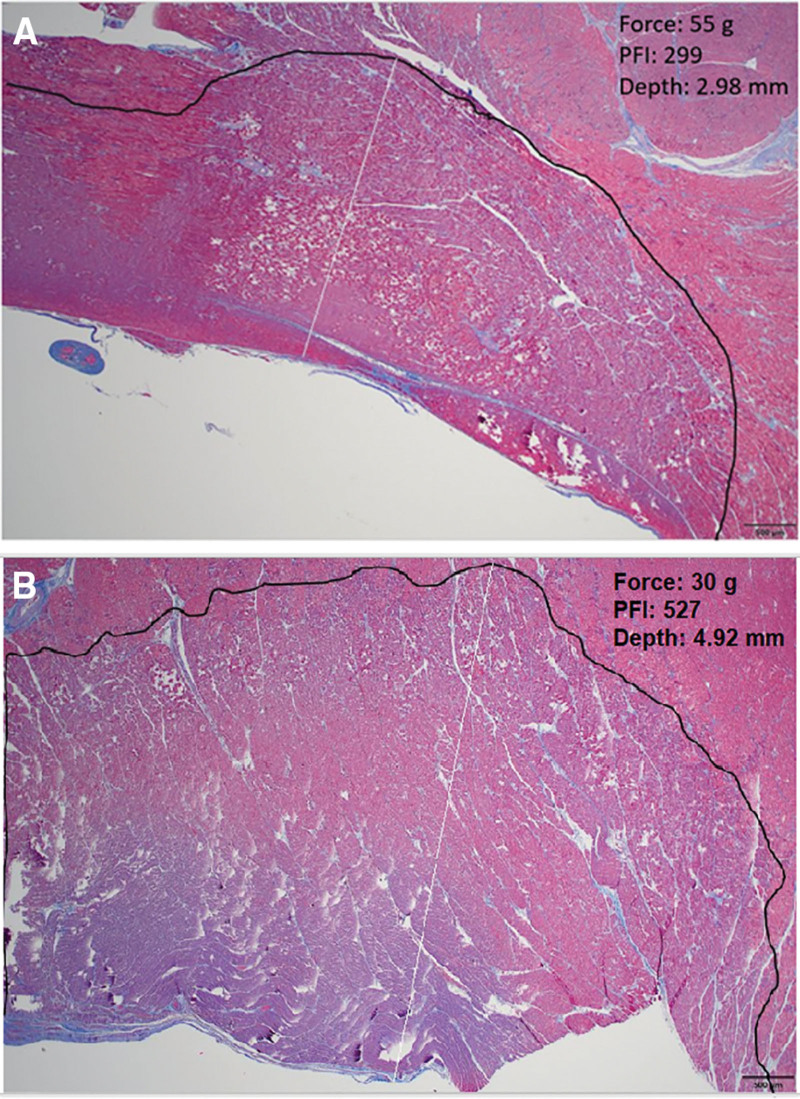
**The histological correlation between pulsed field ablation index (PF index, PFI) and the ventricular lesion depth.** As reported in 2 different specimens, the PF index was clearly correlated with the average ventricular lesion depth. For instance, a PF index of 298 (**A**) and 527 (**B**) was associated with a mean ventricular lesion depth of 2.98 (**A**) and 4.92 (**B**) mm, respectively, thus providing invaluable insights on the prediction of the lesion depth through the PF index value. Images courtesy of © Biosense Webster, Inc. All rights reserved.

PF index and CF were weakly linearly related (Figure 4), and a multiparametric regression analysis was, therefore, tested. We observed that higher PF index values (300, 450, and 600) were associated with progressively deeper lesions (2.86±0.67, 3.92±0.73, and 4.41±0.94 mm, respectively; *P*<0.001). Moreover, the association between PF index and lesion depth was best described by a linear regression model (y=0.923+0.640x; r2=0.66; *P*<0.001; Figure 5A) where the actual ventricular lesion depth could be predicted for each lesion with an accuracy of ±2 mm by dividing the attained PF index by a factor of 100 (Figure 5B). An example of this kind is reported in Figure 6A and 6B on histology specimens.

## DISCUSSION

While the role of CF is clear for RF ablation, data on PFA are scarce. Although Howard et al^3^ demonstrated that biphasic, bipolar PFA delivered through a prototype catheter can form cardiac lesions even in the absence of catheter-tissue contact, some contact seems, nonetheless, required to achieve deeper lesions during PFA delivery. In this regard, Mattison et al^4^ investigated a prototype of a focal PFA catheter in a model of perfused porcine hearts to assess the impact of CF on the ventricular lesion depth. For each PFA application, the CF was held between 1.3 and 48.6 g. The authors found an increase of only 0.01 mm in lesion depth for each additional gram of CF, thus concluding that CF had minimal effects on acute lesion dimension in an isolated porcine heart model undergoing biphasic focal PFA. However, the authors analyzed an ex vivo perfused heart model where PFA applications were delivered in a perpendicular fashion on the epicardial surface only and guided by a mechanical micromanipulator. Moreover, the investigators were blinded to the PFA dose delivered by the system, and no relationship between PFA dose and ventricular lesion depth was analyzed.

In contrast, more recent evidence would point toward a key role of CF in creating adequate lesion depth in the ventricular myocardium. In an in vivo swine model where biphasic low-dose PFA (×12 applications) and high-dose PFA (×24 applications) were delivered at the ventricular myocardium under 3-dimensional mapping guidance and maintaining CF values between 4 and 58 g, Nakagawa et al^2^ showed that lesion depth increased significantly with increasing CF, shifting from 4.9 to 5.8 mm for low- and high-dose PFAs, respectively (*P*=0.01). Furthermore, the slope of the nonlinear correlation equation was different between low- and high-dose PFAs for which the authors suggested the role of different degrees of CF contribution in lesion formation. However, because the focus of the investigators was primarily on the role of CF—and not of PFA dose—on lesion depth, the deeper lesion could, in fact, have been achieved by toggling from low-dose PFA to high-dose PFA regardless of CF values.

To our knowledge, this study represents the first evidence of the impact of CF and PFA dose on the ventricular lesion size in swine undergoing PFA using the CF-sensing OMNYPULSE catheter. Although both variables proved to be involved in adequate lesion formation, more than CF and the number of PFA applications alone, it was their interplay to synergistically act on lesion formation. In fact, once adequate catheter-tissue contact was achieved (CF >5 g), compared with low-dose PFA, high-dose PFA led to remarkably deeper lesions with no clear effect on lesion width. The validation of these parameters through the new PF index formula further helped us understand the impact of CF and the PFA dose on lesion size by predicting the actual ventricular lesion depth. These results are even more important in light of the lack of data on the optimal ablation parameters needed to achieve adequate lesion formation during ventricular tachycardia ablation. In fact, unlike the field of AF ablation where the ablation index algorithm has been extensively investigated,^5^ there is limited experience in the implementation of AI during ventricular tachycardia ablation, and an equivalent index of lesion quality has not been hitherto investigated for PFA in this setting.

However, several study limitations should be acknowledged. First, as none of the evaluated lesions was transmural, further evidence is required to identify not only the best PF index cutoff allowing for the creation of effective and durable lesions but also to investigate whether the PF index can even guide PFA ablation in the atrial chamber as well. In this regard, we observed that the 600 PF index range could not be attained during ablation, and features related to the geometry of the OMNYPULSE catheter and the deployment of PFA in a bipolar versus unipolar fashion might well explain these findings. Second, no chronic assessment was performed, thus limiting the analysis to acute PFA lesions with no data on lesion modification over time. Third, the impact of CF/PFA on lesion depth was only evaluated in the healthy ventricular myocardium with no histological assessment in the diseased myocardium. Fourth, PFA was only delivered through the OMNYPULSE catheter using prespecified ablation parameters. Therefore, it is to be ascertained whether these study results can be extended to other catheter design and energy parameters. Finally, none of the PFA applications was performed with CF <5 g. In this regard, although recent evidence^4^ showed that none or minimal detectable lesions would result from PFA without good catheter-tissue contact and that an increase of 1–2 mm in distance of the catheter tip from the myocardial tissue would lead to a doubling of the energy necessary to create a 3-mm deep lesion,^3^ further studies are required to investigate the best PFA settings to achieve safe and durable lesions for the treatment of cardiac arrhythmias.

## CONCLUSIONS

As assessed on histology, the combined effect of PFA dose and CF during PFA provides a synergistic impact on the ventricular lesion size in swine. The PF index—a new parameter of lesion quality implementing PFA dose and CF—seems to predict the actual lesion size in these animals. Further studies are required to evaluate the best combination of PFA dose and CF values to achieve transmural lesions in both atria and ventricles.

## ARTICLE INFORMATION

### Sources of Funding

This study was supported by Biosense Webster.

### Disclosures

Dr Di Biase is a consultant for Stereotaxis, Biosense Webster, Boston Scientific, Abbott Medical, and I-Rhythm and has received speaker honoraria/travel from Medtronic, AtriCure, Biotronik, Baylis Medical, and Zoll. Dr Hsu has received honoraria from Medtronic, Abbott, Boston Scientific, Biotronik, Janssen Pharmaceuticals, Bristol-Myers Squibb, Pfizer, Sanofi, Zoll Medical, AltaThera, iRhythm, Acutus Medical, Galvanize Therapeutics, vizAI, and Biosense Webster, research grants from Biotronik and Biosense Webster, and has equity interest in Vektor Medical. V. Grupposo, C. Beeckler, and Drs Sharma and Govari are employees at Biosense Webster. The other authors report no conflicts.

## Supplementary Material

## References

[1] Stewart MT Haines DE Miklavčič D Kos B Kirchhof N Barka N Mattison L Martien M Onal B Howard B. Safety and chronic lesion characterization of pulsed field ablation in a Porcine model. J Cardiovasc Electrophysiol. 2021;32:958–969. doi: 10.1111/jce.14980 33650743 10.1111/jce.14980 PMC8048690

[2] Nakagawa H Farshchi S Maffre J Sharma T Govari A Beekler C Altmann A Ikeda A Sugawara M Hussein AA. Effects of electrode contact force on lesion size produced by pulsed field ablation. Heart Rhythm. 2023;20:S92. doi: 10.1016/j.hrthm.2023.03.394

[3] Howard B Verma A Tzou WS Mattison L Kos B Miklavčič D Onal B Stewart MT Sigg DC. Effects of electrode-tissue proximity on cardiac lesion formation using pulsed field ablation. Circ Arrhythm Electrophysiol. 2022;15:e011110. doi: 10.1161/CIRCEP.122.011110 36166690 10.1161/CIRCEP.122.011110 PMC9584049

[4] Mattison L Verma A Tarakji KG Reichlin T Hindricks G Sack KL Önal B Schmidt MM Miklavčič D Sigg DC. Effect of contact force on pulsed field ablation lesions in porcine cardiac tissue. J Cardiovasc Electrophysiol. 2023;34:693–699. doi: 10.1111/jce.15813 36640426 10.1111/jce.15813

[5] Di Biase L Monir G Melby D Tabereaux P Natale A Manyam H Athill C Delaughter C Patel A Gentlesk P; SURPOINT Postapproval Trial Investigators. Composite index tagging for PVI in paroxysmal AF: a prospective, multicenter postapproval study. JACC Clin Electrophysiol. 2022;8:1077–1089. doi: 10.1016/j.jacep.2022.06.007 36137711 10.1016/j.jacep.2022.06.007

