# Supplementary material for: Pulsed Field Ablation Index–Guided Ablation for Lesion Formation: Impact of Contact Force and Number of Applications in the Ventricular Model
Source: Circ Arrhythm Electrophysiol. 2024 Feb 23;17(4):e012717. doi: 10.1161/CIRCEP.123.012717 (PMC11017832; doi:10.1161/CIRCEP.123.012717)
Supplement: Supplementary file 1 [file hae-17-e012717-s001.pdf]

SUPPLEMENTAL MATERIAL

Supplemental Table 1. Part 1: Contact Force and Lesion Dimensions. Actual ablation numbers per parameters.

	PFA applications				
CF range	3X	6X	9X	12X	Subtotal
5-25g	11	11	11	11	44
26-50g	11	12	13	12	48
>51 g	5	5	3	6	17
Subtotal	27	28	27	29	111

Supplemental Table 2. Part 2: PF index evaluation. Actual ablation numbers per parameters.

	PF index			
CF range	300	450	600	Subtotal
5-25g	15	12	2	29
26-50g	12	10	8	30
51-80g	2	1	11	14
Subtotal	29	23	21	73

Supplemental Table 3. Mean number of PFA applications and CF values associated with PF index

		PFA Applications			Force (g)		
**PF_index**	**Count of ablations**	**mean**	**Range**	**std**	**mean**	**range**	**std**
**300**	29	4.7	3-7	1.2	25.7931	4-56	15.73072
**450**	23	10.3	8-12	1.5	31.47826	7-92	20.72081
**600**	21	11.9	11-12	0.2	45.52381	14-75	15.96126
